# A Type IV Extension Supracondylar Fracture With Comminuted Fracture Pattern: A Case Report

**DOI:** 10.5435/JAAOSGlobal-D-23-00020

**Published:** 2023-09-06

**Authors:** Edward Abraham, Abhishek Deshpande, Asher Lichtig, Lucas Paladino

**Affiliations:** From the Department of Orthopaedics, University of Illinois at Chicago, Chicago, IL.

## Abstract

**Case:**

A three year, 11 month old girl sustained a right displaced extension supracondylar fracture (ESF) of the humerus with comminution of the lateral column after an indoor fall. At surgery, fracture reduction showed multidirectional instability. Adequate reduction was achieved by applying longitudinal traction of the arm with partial elbow flexion and forearm supination. One percutaneous medial pin, followed by one lateral cross pin, was used to immobilize the fracture. Normal posterolateral new periosteal bone formation was seen on radiograph on the lateral side. At 5-year follow-up, she had full range of asymptomatic and symmetrical elbow motion.

**Conclusion:**

This case report shows a displaced ESF with a comminuted lateral humeral column, which contributed to a lack of adequate lateral pin purchase on bone. A modified pin fixation technique first with a medial pin and followed by a lateral pin with both placed through the medial column was used for stable fracture fixation. In addition, this case showed that fracture comminution was a contributory factor to the rare multidirectional instability of the Gartland Type IV fracture.

The extension supracondylar fracture (ESF) of the humerus is the most common elbow fracture in children between 3 and 7 years.^1^ In 1959, Gartland reported on a classification system based on three fracture types.^2^ Type I was nondisplaced and stable; type II was partially displaced and partially stable; and type III was completely displaced and unstable. Since then, there have been two important modifications to the classification.^3,4^ In 2006, a rare type IV fracture was suggested as a variant to the Gartland type III fracture.^4^ This new fracture type was described as a multidirectional instability fracture, which can be reduced using a different technique. In their retrospective review of nine cases, the authors thought that the instability of the fracture at the time of reduction was likely because of an incompetent periosteal hinge which may have occurred at the time of injury or by the treating surgeon who converted it to a flexion ESF type.

Other authors have associated this fracture pattern with increased elbow valgus deformity, different fracture lines, lateral translation, and increased flexion angulation of the distal fragment as seen with the flexion-type supracondylar fractures.^5-7^ However, these reports did not attribute the unstable nature of the fracture to the fracture comminution. In addition, there has been a lack of literature on optimal pin fixation of this type IV ESF, with previous studies suggesting that lateral wire fixation may reduce iatrogenic nerve lesions but that crossed wire fixations may provide a more stable configuration for type II and type III fractures.^8-10^

In our case, we are reporting a type IV supracondylar fracture with radiographic evidence of fracture comminution and with findings of an intact posterior lateral periosteum, successfully treated with two crossed pins. This case report suggests that the pattern of fracture comminution may be a crucial factor responsible for causing its instability.

## Case Report

**Figure 1:**
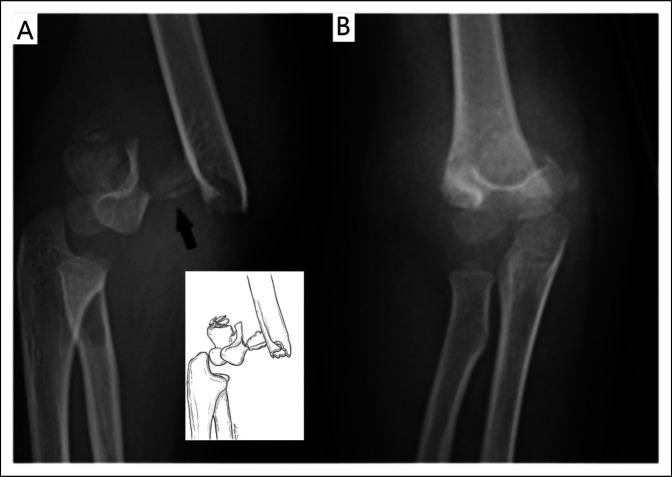
Preoperative radiographs of the right elbow of a displaced and comminuted extension supracondylar fracture of the humerus. **A,** Lateral view shows a large displaced bony fractured fragment (arrow). Insert illustrates a line drawing of the fracture pattern. **B,** Anteroposterior image shows an increased valgus alignment of the elbow.

A 3 year, 11-month-old girl presented to the hospital emergency department where she was diagnosed with a closed, displaced extension (ESF) of the right humerus (type III). The isolated injury was caused when she fell indoors off a sofa at home while playing with her sister. On physical examination, the right elbow area was deformed, swollen, and painful. The right distal radial pulse was absent, but there was normal capillary blood flow to a warm hand. The limited neurologic examination and general examination were normal. Right elbow radiographs showed a comminuted ESF (Figure 1). The upper extremity was splinted with the elbow joint in mild flexion. Eight hours after admission, she underwent a successful closed reduction and crossed percutaneous pin fixation of the fracture. Two unsuccessful attempts were made to reduce and fix the fracture using the standard acute flexion maneuvers and two laterally placed Kirschner 0.62-inch pins. At that point in time, it became clear that the fracture was a multidirectional unstable fracture type, which was caused by a large, displaced fracture fragment from the lateral column of the distal humerus. A different fracture reduction maneuver technique was then used with the arm positioned between the fluoroscopic arms.

**Figure 2:**
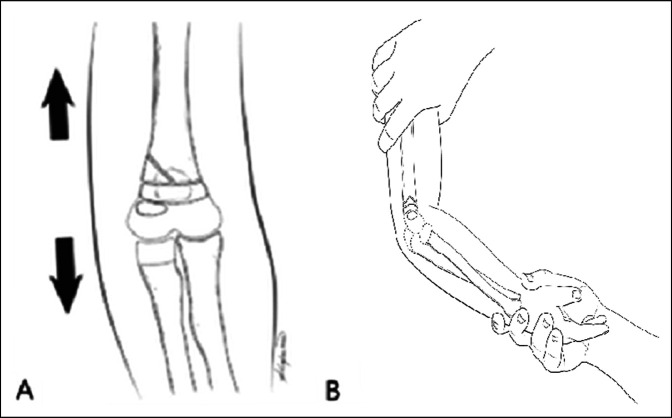
Illustrations showing the fracture reduction technique. **A,** Longitudinal traction (arrows) applied to the extremity to restore fracture reduction and alignment. **B,** The elbow partially flexed at around 45° and the forearm held in a supinated position.

The extremity was extended at the elbow joint to approximately 45 degrees of flexion, and longitudinal traction was applied across the fracture with the forearm supinated (Figure 2). Gentle local multidirectional manipulation of the fracture helped improve its alignment. A lateral radiograph was then obtained to confirm fracture reduction by rotating the C-arm of the fluoroscan around the supported and immobilized extremity. A two centimeter incision was made over the medial epicondyle. A hemostat was used to probe the wound down to bone to ensure that the ulnar nerve was not at risk of injury. A Kirschner pin was drilled from the medial epicondyle, and the wire was then used as a joystick to improve the fracture reduction. The pin was advanced across the fracture and through the lateral humeral cortex. A second percutaneous cross-fixing wire was then drilled from the lateral epicondyle while avoiding the broken lateral humeral column. At that point, the fracture fixation was considered stable. A long arm posterior splint was applied to the arm with the elbow joint flexed to 70°. The hand remained warm and with good capillary circulation but with an absent distal radial pulse. The patient was discharged with postoperative instructions on the same day.

**Figure 3:**
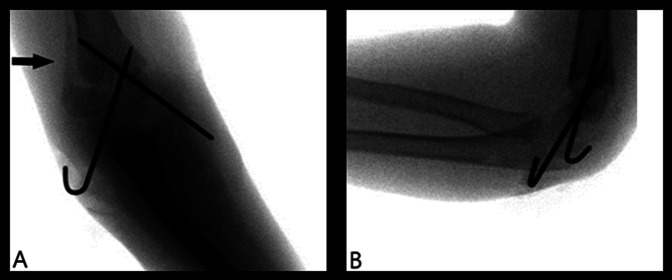
Postoperative radiographs showing cross-pin fixation of fracture at 4 weeks. **A,** Anteroposterior view shows exposed normal posterior lateral periosteal bone formation (arrow). B. Lateral view.

The patient's postoperative follow-up was uneventful. At the first 2-week visit, she had the return of a normal radial pulse. A long arm cast replaced the splint. At her 4-week visit, radiographs showed a healing fracture in satisfactory alignment (Figure 3). The anteroposterior radiograph showed the appearance of normal posterior periosteal new bone formation on the lateral side. The patient's pins were removed, and the cast was discontinued. The patient was then lost to follow-up.

**Figure 4:**
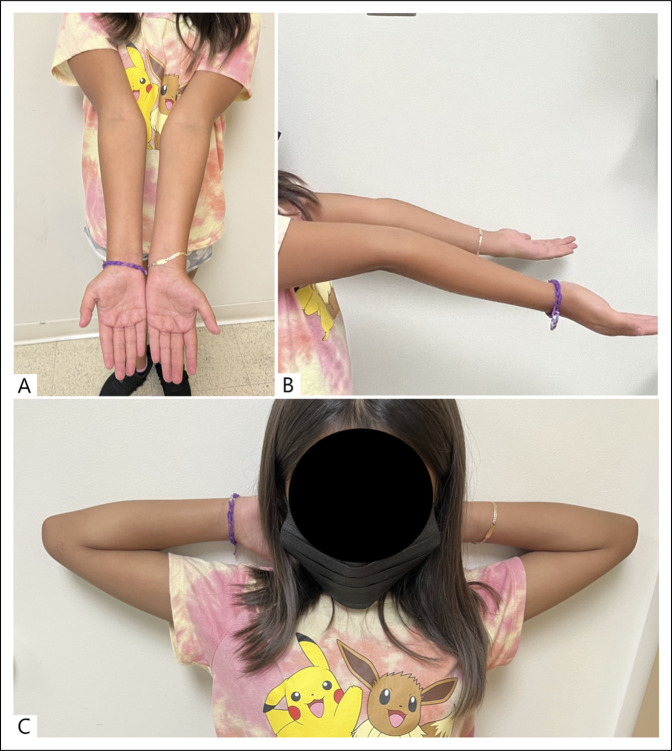
Five-year follow-up of clinical photographs of the patient showing normal and symmetrically equal elbow motion. **A,** Carrying angle 10°. **B,** Elbow flexion 120°. **C,** Elbow extension 14°.

**Figure 5:**
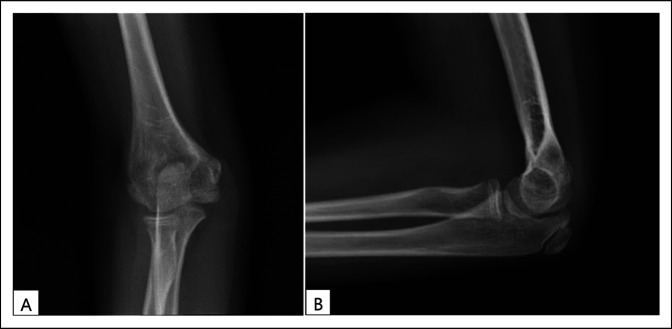
Follow-up radiographs of the right elbow at 5 years. **A,** Anteroposterior view. B. Lateral view.

She was seen in the clinic again after 5 years. She was asymptomatic and had a full range of bilateral symmetrical elbow motion (Figure 4). Elbow radiographs showed a healed and remodeled fracture with normal bone growth and restoration of the lateral humeral column (Figure 5). Both elbows flexed to 120°. She had 14 degrees of hyperextension in the elbows. She had bilateral carrying angles of 10° valgus. The patient's guardians provided informed consent for this case to be published.

## Discussion

This rare case report showed that a displaced ESF of the humerus with comminution of the lateral column and multidirectional instability can be successfully treated by using a modified reduction technique and percutaneous cross-pin fixation which avoided the comminuted lateral column. Satisfactory fracture reduction was obtained by applying longitudinal traction to the extremity, with the elbow partially flexed around 45° and with the forearm supinated. Variations of this ESF with associated intra-articular extension into the elbow joint requiring modified reduction or fixation methods have been reported.^11^

Another important feature of our case was the radiographic visualization of exposed normal posterolateral periosteal new bone formation crossing the fracture with a medially displaced distal humerus. This case report suggests that the fracture comminution of a type III ESF in a child may be an additional major factor contributing to the fracture's multidirectional instability. In the report by Leitch et al^4^ of nine patients treated by closed reduction, they hypothesized that the fracture instability was because of an incompetent periosteal hinge.

The unstable nature of the fracture has been reported by others and also attributed to a grossly ineffective periosteum.^4-7,12^ These authors treated the fractures by mainly closed reduction and percutaneous pin fixation, and they did not report direct or radiographic visualization of the condition of the periosteum during the acute and subacute fracture healing period. Currently accepted hypotheses suggest that the multidirectional fracture instability is because of a torn periosteal hinge or more extensive periosteal disruption. However, our case showed posterolateral periosteal new bone formation with a normal periosteal bridge crossing the medially displaced fracture pattern at 4 weeks after surgery. Fracture comminution may play an important role in the development of this fracture type.

The attempt to classify this multidirectional unstable fracture as a Gartland type IV fracture raised several issues. Because diagnosis of the fracture can only be made at the time of fracture reduction, preoperative treatment planning cannot be based on the new proposed classification. This fracture type is reported to be seen in both extension and flexion-type supracondylar fractures, which have opposite mechanisms of injury and soft-tissue damage.

We estimate that the frequency of the type IV ESF in children and adolescents is approximately 0.9%. This figure is based on an institutional review board–approved data collection log for all upper extremity injuries recorded between July 2011 and October 2021 from the principal investigator's (EA) clinical practice. This case report was the only patient who fit this diagnosis from a total 111 ESF patients.

This case report described a multidirectional, unstable proposed Gartland type IV extension supracondylar fracture in a young child. The comminuted fracture was associated with a large free bone fragment from the lateral humeral column and exposed normal periosteal bone formation crossed the fracture from the posterolateral side. The fracture was successfully treated by a modified closed reduction maneuver and two cross-pin fixation which avoided the lateral column.

## References

[1] Omid R Choi PD Skaggs DL: Supracondylar humeral fractures in children. J Bone Joint Surg Am Vol 2008;90:1121-1132 10.2106/JBJS.G.01354 18451407

[2] Gartland JJ: Management of supracondylar fractures of the humerus in children. Surg Gynecol Obstet, Philadelphia, PA, USA 1959;109:145-154. 13675986

[3] Wilkins KE: Fractures and dislocations of the elbow region, in Rockwood C, ed: Fractures in Children. JB Lipppincott, 1984, pp 363-575.

[4] Leitch KK Kay RM Femino JD Tolo VT Storer SK Skaggs DL: Treatment of multidirectionally unstable supracondylar humeral fractures in children. A modified Gartland type-IV fracture. J Bone Joint Surg 2006;88:980-985 16651572 10.2106/JBJS.D.02956

[5] Sharma A Sethi A: Multidirectionally unstable supracondylar humeral fractures in children. JBJS Rev 2019;7:e3 10.2106/JBJS.RVW.18.00045 30870315

[6] Mitchell SL Sullivan BT Ho CA Abzug JM Raad M Sponseller PD: Pediatric Gartland type-IV supracondylar humeral fractures have Substantial overlap with flexion-type fractures. J Bone Joint Surg 2019;101:1351-1356 31393425 10.2106/JBJS.18.01178 PMC7406141

[7] Barik S Singh G Maji S Azam MQ Singh V: Preoperative prediction of Gartland IV supracondylar fractures of humerus: Is it possible? Rev Bras Ortop 2021;56:230-234 10.1055/s-0040-1722578 PMC8075635 33935319

[8] Carrazzone OL Barbachan Mansur NS Matsunaga FT : Crossed versus lateral K-wire fixation of supracondylar fractures of the humerus in children: A meta-analysis of randomized controlled trials. J Shoulder Elbow Surg 2021;30:439-448 33069907 10.1016/j.jse.2020.09.021

[9] Hamdi A Poitras P Louati H Dagenais S Masquijo J Kontio K: Biomechanical analysis of lateral pin placements for pediatric supracondylar humerus fractures. J Pediatr Orthop 2010;30:135-139 20179560 10.1097/BPO.0b013e3181cfcd14

[10] Na Y Bai R Zhao Z : Comparison of lateral entry with crossed entry pinning for pediatric supracondylar humeral fractures: A meta-analysis. J Orthop Surg Res 2018;13:68 29615086 10.1186/s13018-018-0768-3 PMC5883290

[11] Abraham E Gordon A Abdul-Hadi O: Management of supracondylar fractures of humerus with condylar involvement in children. J Pediatr Orthop 2005;25:709-716 16294122 10.1097/01.bpo.0000184645.96356.fe

[12] Soldado F Hodgson F Barrera-Ochoa S : Gartland type-IV supracondylar humeral fractures: Preoperative radiographic features and a hypothesis on causation. Orthop Traumatol Surg Res 2022;108:103049 34500111 10.1016/j.otsr.2021.103049

